# The microbial communities in *Zaopeis*, free amino acids in raw liquor, and their correlations for *Wuliangye‐flavor* raw liquor production

**DOI:** 10.1002/fsn3.2872

**Published:** 2022-04-08

**Authors:** Bin Jiang, Li Wu, Qi Wang, Liran Yang, Jia Zheng, Shulai Zhou, Cuirong He, Wenwen Jiao, Bin Xu, Kunyi Liu

**Affiliations:** ^1^ College of Wuliangye Technology and Food Engineering & College of Modern Agriculture Yibin Vocational and Technical College Yibin China; ^2^ Leshan Food and Drug Inspection Center Leshan China; ^3^ Flavor Innovation Center Technology Research Center, Wuliangye Yibin Co., Ltd. Yibin China; ^4^ Department of Pharmacy Leshan Vocational and Technical College Leshan China; ^5^ Sichuan Research Institute of Alcoholic Drinks Chengdu China; ^6^ Luzhou Greenland Wine Co., Ltd. Luzhou China

**Keywords:** correlation, free amino acid, microbial community, *Wuliangye‐flavor* raw liquor, *Zaopei*

## Abstract

*Wuliangye‐flavor* liquor (WLFL), a specific Chinese traditional liquor, one of the major type of global distilled spirits, offers a unique flavor system acquired across thousands of years of development. Free amino acids (FAAs), as major health factors, are considered to be primarily derive from the hydrolysis of protein from the *Zaopeis* (ZPs) by microbial populations during fermentation. Here, we investigated the changes of microbial communities in ZPs and FAAs in raw liquor (RL) directly related ZPs from different ages of WLFL fermentation pits by phospholipid fatty acid fingerprint (PLFA) and high performance liquid chromatography method. Results indicated that the total PLFAs of 20‐ and 50‐year ZPs were significantly higher (*p* < .05) than 1‐ and 5‐year ZPs. Gram‐positive bacteria (G^+^), anaerobic bacteria, and fungi were dominant in the ZPs. Furthermore, the total of FAAs content was highly increased (*p* < .05) in RLs of aged fermentation pit (20‐ and 50‐year, 24.86–30.23 mg/g, 28.73–37.15 mg/g) compared with young (1‐ and 5‐year, 20.97–26.46 mg/g, 17.83–28.70 mg/g), while, the different ages of RLs could be distinguished according to 9 biomarkers of FAAs (Variable importance in projection, VIP >1; *p* < .05), including tyrosine, aspartic acid, leucine, glutamic acid, citrulline, alanine, proline, glycine, and valine. Particularly, the biomarkers of FAAs were positively correlated with gram‐negative bacteria (G^−^) and fungi, but negatively correlated with G^+^. This is the first report to uncover the microbial communities in *zaopeis*, free amino acids in RL, and their correlations for *Wuliangye‐flavor* raw liquor production.

## Graphical abstract

The total biomass, fungal biomass, and the biomass of G^+^ bacteria increased as the age of the pit increased for *Wuliangye‐flavor* raw liquor production. The biodiversity of microorganisms in the *zaopeis* of aged fermentation pit and lower layer higher than the young fermentation pit and upper layer. The differential free amino acids were positively correlated with G^−^and fungi, while negatively correlated with G^+^.

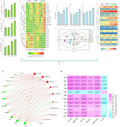

## 1 INTRODUCTION

Chinese liquor (Baijiu), approximately 40%–60% alcohol by volume, is one of the world's four most popular distilled spirits along with whisky, brandy, and vodka (Huang et al., 2017). Interestingly, Chinese liquor is classified primarily as either strong‐flavor (also called thick favor or *Luzhou* favor) liquor and Jiang‐flavor (also called sauce favor) liquor according to its distinctive favor and taste (Huang et al., 2017; Xu et al., 2010). Strong‐flavor liquor is one of the most important traditional distillates in China and can be distinguished from other Chinese liquors such as Moutai and Fen liquor based on its production process (Zhao et al., 2012). Due to the historical and cultural factors, this type of liquor plays a particular role in Chinese traditional culture, and it is now very popular in China and several other countries (Xu et al., 2017; Zheng et al., 2013). *Wuliangye‐flavor* liquor (WLFL), also known as strong flavor liquor, is one of the main Chinese distilled spirits and has a history of hundreds of year (Liu et al., 2017; Wang et al., 2021). It also has been awarded the distinction of “National Famous Liquor” four out of five times (Shen, 2007). WLFL requires five grains (sorghum, glutinous rice, rice, wheat, and corn) as raw materials, namely, *Zaopeis* (ZPs), is brewed in a unique natural environment (Wang et al., 2021). The production process for WLFL usually takes approximately many months. Briefly, fresh grains are mixed with the fermented grains and steamed rice husks for distillation; after the distillation completed, the cooled distilled mixture is mixed with *Daqu* (a fermentation starter); the mixture is placed into the pit and fermented for 60–90 days; then the fermented grains are sequentially processed in the way described above (Wang et al., 2021; Zheng et al., 2013).

In fact, the special flavor of WLFL can be affected by the raw materials, *Daqu*, fermentation, distillation, blending, aging, microbial, etc., and among these, microorganisms involved in the fermentation process are vital for the quality characteristics formation of WLFL (Liu & Sun, 2018; Tan et al., 2019). It is widely believed that ZPs are the specific fermentation matrix that microorganisms inhabit, provides nutrition(Li et al., 2011; Zhang et al., 2016), and support the formation of microbial metabolites, especially the volatile compounds (Zhang et al., 2005). Meanwhile, starch, protein, and other macromolecules are degraded and utilized during fermentation, resulting in the accumulation of water, amino acids, ethanol, acids, and other compounds in ZPs (Gao et al., 2020). Meanwhile, during the fermentation of microorganisms to generate liquor flavor substances, amino acids are the main source of assimilable nitrogen (Liu et al., 2018), and somehow they determine the microbial growth and the production of metabolites (Procopio et al., 2015; Simoné, 2015). More importantly, some free amino acids (FAAs), such as leucine, isoleucine, valine, proline, glutamine, and cysteine can influence the aroma and even the flavor of the final liquor (Garde‐Cerdán & Ancín‐Azpilicueta, 2008; Procopio et al., 2015; Zheng et al., 2012). Further, more and more consumers have concerns about the nutrition and health effects of Chinese liquor (Huo et al., 2020). Whereas, many bioactive compounds (also called health factors) in Chinese liquor are found to be beneficial to human health, such as FAAs, phenols, acids, pyrazine, peptides, etc., which have the functions of promoting ethanol metabolism, improving the comfort after drinking, anti‐oxidation, anti‐inflammatory, anti‐cancer, and prevention and treatment of cardiovascular diseases (Liu et al., 2020; Wu et al., 2017). Besides, as the main raw material for commercial WLFL, *Wuliangye‐flavor* raw liquor (WLFL‐RL) plays an important role in the formation of quality characteristics. Therefore, it was necessary to reveal the correlation between FAAs of WLFL‐RLs and microbial communities in ZPs directly related to WLFL‐RLs.

In the latest two decades, culture‐independent approaches were developed and employed to investigate the microbial communities, especially the bacterial community in WLFL's ZPs, and *Lactobacillus acetotolerans* was identified as the main genera in WLFL fermentation (Zhang et al., 2016; Zheng et al., 2013). Previous studies indicated that the phospholipid fatty acid (PLFA) fingerprint technology is a sensitive, rapid and cost‐effective culture‐independent technology was appropriate for characterizing the quantitatively determine microbial compositions in various environmental samples on several levels from the whole community profile to specific group abundance, total biomass, and their shifts with sampling position in a fermentation pit (Ding et al., 2015; Zhao et al., 2012; Zheng et al., 2014). Zheng et al. (2013) and Wang et al. (2021) used it to reveal that gram‐positive bacteria (G^+^), aerobe bacteria and fungi predominated in the ZPs of strong‐flavor liquors. However, few reports have focused on the spatial distribution of microbial communities and their biodiversity in WLFL's ZPs from different fermentation pits.

Consequently, this study aimed to investigate the FAAs in WLFL‐RLs of different fermentation pit ages by high performance liquid chromatography (HPLC) and the relationship between FAAs and microbial communities in various ZPs directly corresponding to WLFL‐RLs. Multivariate statistical techniques were used to investigate biomarkers of FAAs components of WLFL‐RLs in different fermentation pit ages and to understand the correlation between biomarkers of FAAs and microbial communities in ZPs. To the best of our knowledge, this is the first report to uncover the microbial communities in *zaopeis*, free amino acids in RL, and their correlations for WLFL‐RL production. The results can be used as reference to understand the correlation between FAAs and microbial communities of WLFL‐RL in the later stage and broaden the application range of health factors.

## 2 MATERIALS AND METHODS

### 2.1 Chemicals and reagents

Citric acid, sodium citrate, chloroform, acetone, toluene, acetic acid, potassium hydroxide, trichloroacetic acid, orthohexane, sodium acetate, acetic acid, methanol, acetonitrile and tetrahydrofuran were purchased from Chengdu Cologne Chemical Reagent Factory. The standards (≥98.0%) of glutamate (Glu), aspartate (Asp), citrulline (Cit), threonine (Thr), glycine (Gly), arginine (Arg), serine (Ser), methionine (Met), leucine (Leu), proline (Pro), isoleucine (I‐leu), alanine (Ala), tyrosine (Tyr), cysteine (Cys), valine (Val), histidine (His), phenylalanine (Phe), lysine (Lys), and phthalformaldehyde, o‐phthalaldehyde, 9‐fluorenylmethylchloroformate were purchased from Sigma‐Aldrich. Deionized water was used in the EPED‐T purification system (Yi Pu Yi Da Co.) and was degassed under vacuum and filtered through a 0.45 μm nylon membrane before use for HPLC analysis.

### 2.2 Sampling procedure

In order to determine the microbial communities in ZPs of different ages, FAAs in RLs of different ages, and their correlations for WLFL‐RL production, RLs and directly related ZPs were simultaneously collected from a *Wuliangye‐flavor* liquor producing company in Yibin, Sichuan, China in July 2019. 36 RLs in the four fermentation pit from commercial level workshop were obtained; the pit ages were 1, 5, 20, and 50 years; the sample was the heart cut of distillation (head cut were dispensed), and corresponding to them were 36 ZPs, which were collected from the upper layer, middle layer, and lower layer in the same fermentation pit, respectively, and three ZPs sample were collected from each layer (Table 1). Meanwhile, the ZPs were collected by 5‐point sampling methods; approximately 200 g ZPs of each layers were then collected by the ZPs core sampler, and then samples were pooled into sterile stomacher bag, respectively, and stored at −20°C until further analysis; also, the RLs were stored at room temperature.

**TABLE 1:** Serial numbers of the samples

Layers	1 year	5 years	20 years	50 years
Upper	Y1‐U	Y5‐U	Y20‐U	Y50‐U
Middle	Y1‐M	Y5‐M	Y20‐M	Y50‐M
Lower	Y1‐L	Y5‐L	Y20‐L	Y50‐L

Note

Three biological replicates of each layer samples.

### 2.3 Determination of PLFAs

Phospholipid fatty acid fingerprints in ZPs were determined according to the methods reported previously (Zheng et al., 2013, 2014). Each sample was analyzed thrice. Whereas, the microbial community structure were characterized by PLFA, as described previously (Qian et al., 2021; Zelles, 1999).

### 2.4 Determination of FAAs

One mL RL was mixed with 1ml acetocaustin (10%), and centrifuged at 10,000 *g* at room temperature for 15min. Then, the resulting supernatant was filtered through a 0.45 μm nylon membrane and subjected to HPLC (Agilent 1200 series) to determine the FAAs content after precolumn derivatization with *o*‐phthaldialdehyde‐3‐ mercaptopropionic acid (OPA) and 9‐fluorenylmethyl‐chloroformate (FMOC), as described previously (Araque et al., 2013; Mattivi et al., 2000). Each sample was analyzed three times.

### 2.5 Statistical analysis

Data of all groups obtained from gas chromatography (GC) and HPLC analysis were initially processed by one‐way analysis of variance (ANOVA) at the 5% significant level using IBM SPSS Statistics 23.0 (SPSS Inc.) to compare the differences statistically. Heat maps were generated and cluster analyses of the PLFAs and FAAs compounds were obtained with the TBtools software (Shannon et al., 2003), and multivariate data analysis was conducted with SIMCA‐P 14.1 software. Principal component analysis (PCA) was performed to obtain an overview of the sample distribution and observe possible outliers. Orthogonal partial least‐squares discrimination analysis (OPLS‐DA) was performed to identify the metabolites that significantly contributed to clustering and discrimination. S‐plot and variable importance in projection (VIP) were used to evaluate the variable contribution. Variables with VIP >1 and *p* < .05 were deemed as potential biomarkers. A valid co‐occurrence was selected as a strong correlation if the Spearman's correlation coefficient (*ρ*) was >.6 (Barberan et al., 2012). Correlation networks were constructed with the Cytoscape (Rousk et al., 2010).

## 3 RESULTS AND DISCUSSION

### 3.1 Statistical analysis

**FIGURE 1:**
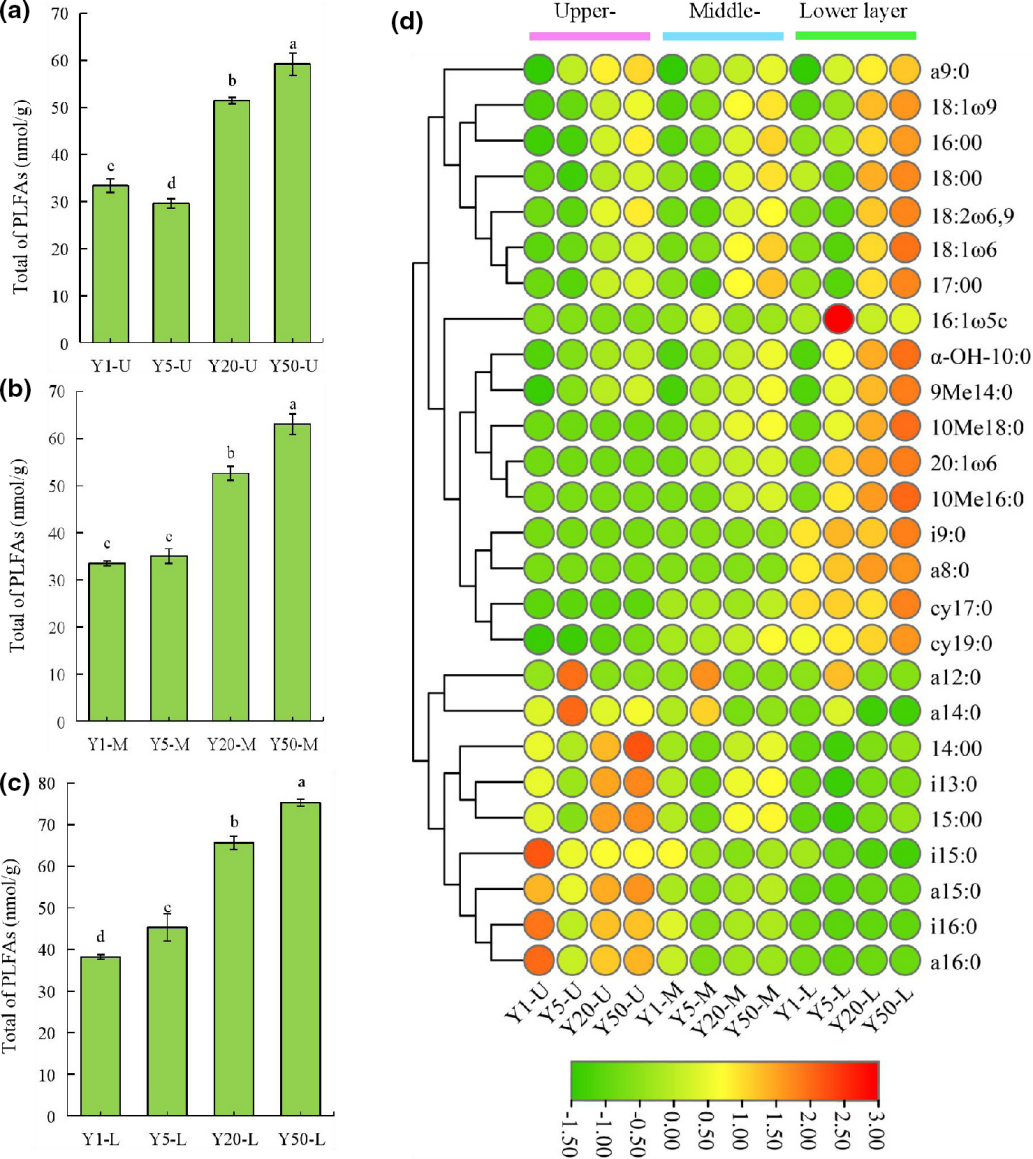
Changes of PLFAs of *Zaopeis* (ZPs) at different fermentation pit ages. Total PLFAs in the Upper‐layer (a), Middle‐layer (b), and Lower‐layer (c). Error bars indicated standard deviations (*n* = 3). Different letters indicated statistical differences from each other (*p* < .05). Heat map of 26 PLFAs in ZPs (d). Y1‐Y50 represents 1‐, 5‐, 20‐, 50‐year ZPs, respectively. U, M, L represents upper‐layer, middle‐layer, lower‐layer ZPs, respectively

Microbial biomass, assessed by total PLFAs, of ZPs with different fermentation pit ages are shown in Figure 1a–c. The contents of total PLFAs in the ZPs of different layers increased correspondingly with the fermentation pit age (from 105.07 to 197.57 nmol/g, Y1–Y50), and these findings were similar to those reported previously (Zhao et al., 2012; Zheng et al., 2014). This may be related to the uninterrupted fermentation that occurred in the cellar for several decades: the microbial communities in the pit mud might have experienced long‐time oxygen deficiency and high acid domestication, and those microorganisms that were not suitable for growth in high ethanol content, low pH, microoxygen environments would have phased out, while microorganisms capable of growth in this extreme environment would have been constantly enriched, making the biodiversity of microorganisms in the ZPs of aged cellar higher than that of the young cellar (Ding et al., 2015; Xu et al., 2017).

As shown in Figure 1d, 26 PLFAs were identified: these PLFAs were compounds ranging from C_5_ to C_30_, and they consisted of five normal saturated PLFAs, four monounsaturated PLFAs, one polyunsaturated PLFA, one OH‐branched PLFA, 13 methyl‐branched PLFAs, and two cyclopropyl PLFAs. As shown in Figure 1a–d and Table S1, in the upper‐layer (U), 50‐year‐ZPs had the highest total PLFAs (*p* < .05), with the level of 59.21 nmol/g, followed by 20‐year‐ZPs: the dominant PLFAs were a16:0, i16:0, a15:0, i15:0, i13:0, 14:0, and 13:0. In the middle‐layer (M), the total PLFAs were increased corresponding to the pit ages, the level from 33.51 to 63.06 nmol/g: the dominant PLFAs were i15:0, a14:0, a12:0, 17:0, 15:0, 18:1ω6, 16:0, and 18:0. In the lower‐layer (L), 50‐year‐ZPs had the highest total PLFAs (*p* < .05), with the level of 75.30 nmol/g, followed by 20‐year‐ZPs. The dominant PLFAs were cy19:0, cy17:0, a8:0, i9:0, 10Me16:0, 10Me18:0, 20:1ω6, 9Me14:0, α‐OH‐10:0, 18:1ω9, and 18:2ω6,9. Simultaneously, the upper layer had the highest total PLFAs (*p* < .05), followed by the middle layer. This indicates that the relative abundance of microorganisms gradually increased from the upper layer to the lower layer. This may be due to the fact that the brewing microorganisms in the fermentation pit produced a large amount of nutrients such as bacterial autolysates, humus, and organic acids during the production process, which gradually penetrated into the yellow water, enabling the more brewing microorganisms to grow and multiply better in bottom of the pit mud and the lower layers of ZPs (Li et al., 2017; Zheng et al., 2013).

**FIGURE 2:**
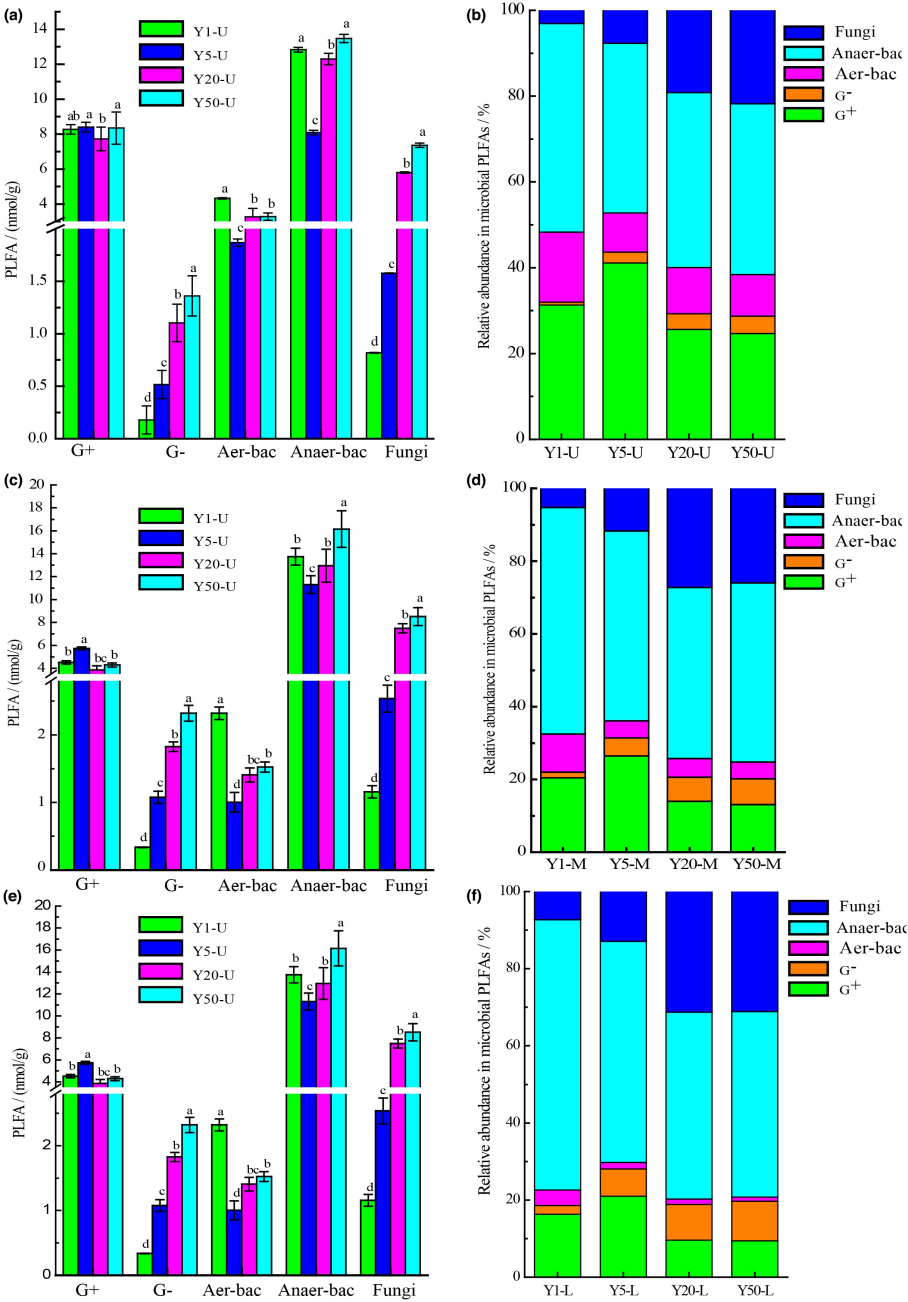
Microbial community structural characteristics in different fermentation pit ages. Content of each type of microorganisms in the upper layer (a), Middle‐layer (c) and Lower‐layer (e). Error bars indicated standard deviations (*n* = 3). Different letters indicated statistical differences from each other (*p* < .05). Relative abundance of microorganisms in the upper layer (b), Middle‐layer (d), and Lower‐layer (f). Y1‐Y50 represents 1‐, 5‐, 20‐, and 50‐year *Zaopeis* (ZPs), respectively. U, M, and L represents upper‐layer, middle‐layer, and lower‐layer ZPs, respectively

Simultaneously, the PLFAs based fingerprinting method could rapidly quantify the information of the living cells, especially the specific group abundance. It revealed that microbial communities in the ZPs were composed gram‐positive bacteria (G^+^) and gram‐negative bacteria (G^−^), anaerobic bacteria (Anaer‐bac), aerobic bacteria (Aer‐bac), as well as fungi. Of them, G^+^, Anaer‐bac, and fungi were dominant in the ZPs, and these findings were in accordance with the results of previous studies conducted using culture‐dependent and culture‐independent technologies (Qian et al., 2021; Zheng et al., 2013). Additionally, the relative abundance of fungi increased with pit ages: this might have occurred owing to the wider pH range for the optimal growth of fungi (Rousk et al., 2010; Zhao et al., 2012). Moreover, G^+^ was highest in 5‐year ZPs, Aer‐bac was highest in 1‐year ZPs, Anaer‐bac was highest in 50‐year ZPs, while G^−^ and fungi were increased with pit ages, and the relative abundance of G^+^ was much higher compared to that of G^−^ in each sample, indicating that bacterial communities in the ZPs was dominated by G^+^. This was in agreement with our previous research results, where G^+^ was the dominant species in the ZPs (Wang et al., 2021) (Figure 2 and Table 2). In the upper‐layer of ZPs, G^−^ and fungi were increased with pit ages, whereas Aer‐bac and Anaer‐bac were initially decreased and then increased, and close to 50‐year ZPs (Table 2 and Figure 2a). The proportion of G^−^ increased from 0.67% to 4.02% (Y1–Y50), fungi increased from 3.10% to 21.77% (Y1–Y50), Aer‐bac decreased from 16.36% to 9.68% (Y1–Y50), and Anaer‐bac decreased from 48.56% to 39.85% (Y1–Y50), respectively (Figure 2a,b). As shown in Figure 2c,d, in the middle‐layer of ZPs, the proportion of G^−^ increased from 1.52% to 7.08% (Y1–Y50), fungi increased from 5.24% to 25.97% (Y1–Y50), Aer‐bac decreased from 10.52% to 4.65% (Y1–Y50), and Anaer‐bac decreased from 62.26% to 49.24% (Y1–Y50), respectively. The microorganisms in the lower layer have also exhibited a similar trend (Figure 2e,f). Besides, the relative abundance of G^+^ and aerobes in different layers in the fermentation pit decreased gradually from the top to bottom (Table 2), and the contents of G^−^, anaerobe, and fungi were found to be in the opposite order (Table 2). Therefore, these results were in accordance with the phenomenon that the oxygen concentration in the fermentation pit decreased gradually from the top to bottom (Zhang et al., 2017). Moreover, a big decrease in the G^+^/G^−^ ratio was found from the top to bottom and with the pit age in the ZPs (Table 2): this did not coincided with the previous study, mainly as a result of environmental disturbances (Feng & Simpson, 2009).

**TABLE 2:** Contents and ratios of microorganisms in different fermentation pit ages of *Zaopeis*

Samples	G^+^ (nmol/g)	G^−^ (nmol/g)	Aer−bac (nmol/g)	Anaer‐bac (nmol/g)	Fungi (nmol/g)	G^+^/G^−^ (%)
Y1‐U	8.26 ± 0.13^b^	0.17 ± 0.01^e^	4.32 ± 0.13^c^	12.82 ± 0.27^a^	0.81 ± 0.03^d^	48.59
Y1‐M	4.51 ± 0.15^b^	0.33 ± 0.01^e^	2.32 ± 0.09^c^	13.73 ± 0.73^a^	1.15 ± 0.09^d^	13.67
Y1‐L	3.31 ± 0.01^b^	0.45 ± 0.01^e^	0.81 ± 0.03^d^	14.20 ± 0.73^a^	1.48 ± 0.09^c^	7.36
Y5‐U	8.39 ± 0.40^a^	0.51 ± 0.03^c^	1.86 ± 0.08^b^	8.08 ± 0.32^a^	1.57 ± 0.09^bc^	16.45
Y5‐M	5.73 ± 0.12^b^	1.07 ± 0.09^d^	1.00 ± 0.14^d^	11.30 ± 0.77^a^	2.53 ± 0.19^c^	5.36
Y5‐L	5.31 ± 0.27^b^	1.79 ± 0.08^d^	0.42 ± 0.14^e^	14.53 ± 0.77^a^	3.27 ± 0.15^c^	2.97
Y20‐U	7.72 ± 0.32^b^	1.10 ± 0.04^e^	3.25 ± 0.18^d^	12.29 ± 0.67^a^	5.79 ± 0.48^c^	7.02
Y20‐M	3.84 ± 0.36^c^	1.82 ± 0.07^d^	1.40 ± 0.10^d^	12.95 ± 1.44^a^	7.49 ± 0.41^b^	2.11
Y20‐L	3.06 ± 0.22^c^	2.96 ± 0.19^c^	0.45 ± 0.10^d^	15.45 ± 1.44^a^	9.96 ± 0.34^b^	1.03
Y50‐U	8.34 ± 0.23^b^	1.35 ± 0.12^e^	3.27 ± 0.19^d^	13.47 ± 0.92^a^	7.35 ± 0.20^c^	6.18
Y50‐M	4.28 ± 0.17^c^	2.32 ± 0.11^d^	1.52 ± 0.08^de^	16.14 ± 1.59^a^	8.51 ± 0.78^b^	1.84
Y50‐L	3.45 ± 0.04^c^	3.75 ± 0.03^c^	0.40 ± 0.07^d^	17.62 ± 1.59^a^	11.38 ± 0.39^b^	0.92

Note

Different lowercase letters indicate significant differences in the content of microorganisms (*p* < .05). Data are presented as the mean ± standard deviation (*n* = 3).

### 3.2 Multivariate statistical analysis based on PLFAs

**FIGURE 3:**
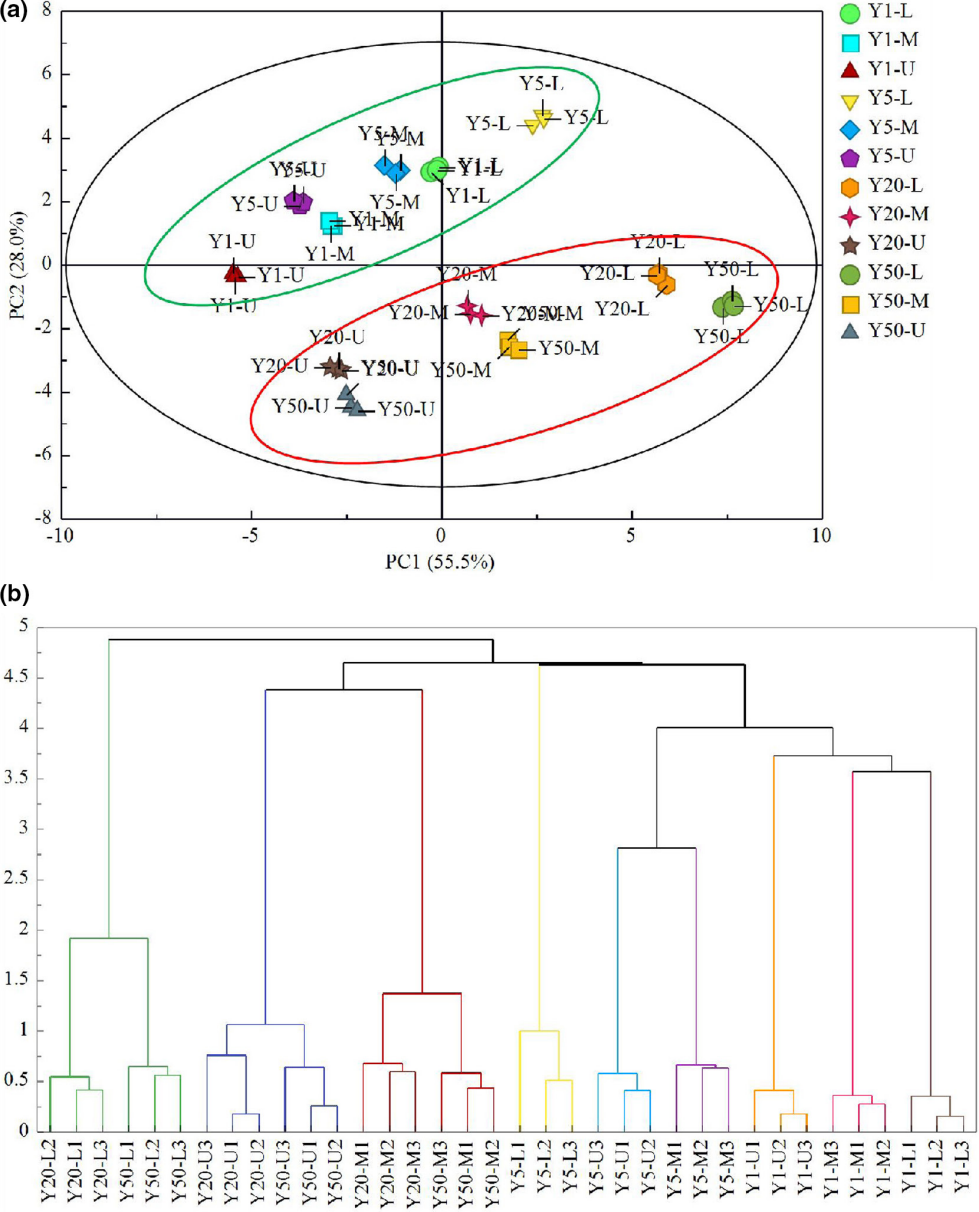
Multivariate statistical analysis based on the concentration of PLFAs. Scores plot of principal component analysis (a). Scores along the two principal components (PC1: 55.5% and PC2: 28.0%) explained 83.5% of the total variability. Results of cluster analysis (b). Y1‐Y50 represents 1‐, 5‐, 20‐, 50‐year *Zaopeis* (ZPs), respectively. U, M, and L represents upper‐layer, middle‐layer, and lower‐layer ZPs, respectively

Principal component analysis is a kind of multivariate statistical analysis method that can be used for the analysis of a database pertaining to several interconnected dependent variables, and it plays well in reducing the dimensionality of the database (Jolliffe, 2005). PCA was performed to comparatively analyze the relationships of the microbial communities in the ZPs of different ages. The score plots are as presented in Figure 3a, the first principal component (PC1) and second principal component (PC2) were taken as coordinate axes for the PCA analysis on samples, and it was noted that the linear combination of PC1 and PC2 explained 83.5% of the total variance of ZPs. As can be seen, the first PC1 explained 55.5% of the total variance and a general separation among ZPs was evidently separated via the loading plot values on PC1 axis. It should be noted that the microbial communities could be clustered into two groups: Group I included young ZPs (Y1 and Y5), and Group II included aged ZPs (Y20 and Y50); this may has been caused by the similarity of PLFA composition in those two ZPs.

In order to realize further the separation among the four ZPs, average values of 26 PLFAs were analyzed by cluster analysis (CA) using Euclidean distance (Xu et al., 2017). All ZPs were classified into three clusters: Y1 and Y5, Y20‐U, Y20‐M and Y50‐U, Y50‐M, Y20‐L and Y50‐L, which meant young ZPs and aged ZPs were well separated from each other (Figure 3b).

To be more specific, both Y20‐L and Y50‐L were visually different from other ZPs in the score plot, indicating that their PLFAs profile was distinct from others. In contrast, the phenotypes of Y20‐U, Y20‐M and Y50‐U, Y50‐M, Y1‐U, M, L, Y5‐U, M and Y5‐L clustered together, respectively. These results were mainly caused by the similar composition of PLFAs in ZPs, with the PCA showing similar clustering.

### 3.3 Analysis of FAAs in RLs of different fermentation pit ages

**FIGURE 4:**
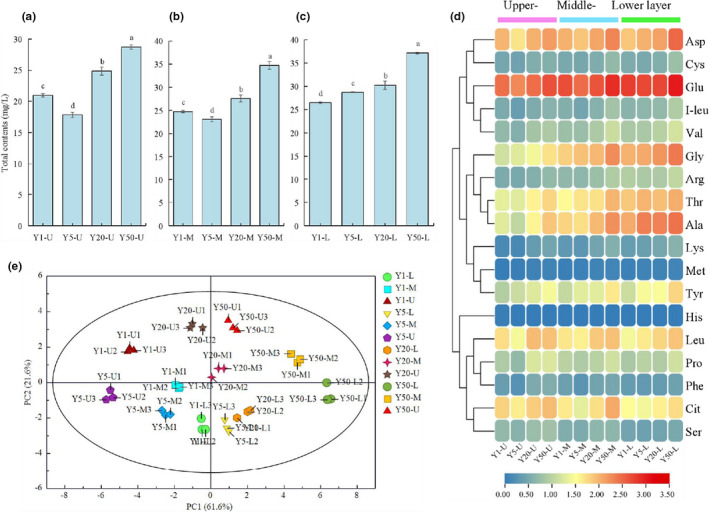
Changes of the contents of the FAAs of RLs at different fermentation pit ages. Total FAAs in the Upper‐layer (a), Middle‐layer (b), and Lower‐layer (c). Error bars indicated standard deviations (*n* = 3). Different letters indicated statistical differences from each other (*p* < .05). Heat map of 18 FAAs in RLs at different pit ages (d). Y1‐Y50 represents 1‐, 5‐, 20‐, and 50‐year RL, respectively. U, M, and L represents upper‐layer, middle‐layer, and lower‐layer RLs, respectively

During the fermentation of microorganisms to generate liquor flavor substances, amino acids are the main source of assimilable nitrogen (Liu et al., 2018), and they are absorbed by microorganisms in a sequential order by a number of amino acid transporters located in the cell membrane, and somehow, this determines the microbial growth and the production of metabolites (Procopio et al., 2015; Simoné, 2015). More importantly, some amino acids are the precursors of aromatic compounds, and they react with carbohydrates at high temperature during the process of making high temperature distilled wine, which contributes to the formation of adequate aromatic compounds that contribute the final taste of Chinese liquor (López‐Rituerto et al., 2010; Procopio et al., 2015). FAAs were detected in RLs from different fermentation pit ages and layers by HPLC analysis. The contents of FAAs significantly differed among different fermentation pit ages (Figure 4a–c and Table S2). The total FAA content was high (*p* < .05) in aged fermentation pit of RLs (Y20 and Y50), compared to young fermentation pit of RLs (Y1 and Y5). Moreover, in lower layers, the total FAA content increased significantly (*p* < .05) in accordance with the pit ages (from 26.46 to 37.15 mg/g). As shown in Figure 4d, 18 FAAs were identified; Glu, Asp, Gly, Thr, Ala, Tyr, Leu, and Cit were the dominant FAAs. In the upper layer RLs of different pit ages, the contents of Gly (*p* = .02), Met (*p* = .002), Tyr (*p* = .01) were increased significantly (*p* < .05) corresponding to pit ages, and their contents were increased from 1.22 to 2.22 mg/g (Y1–Y50), from 0.01 to 0.05 mg/g (Y1–Y50), and from 0.92 to 1.70 mg/g (Y1–Y50), respectively. In the middle layer, the contents of Thr (*p* = .03), Gly (*p* = .02), Met (*p* = .001), Tyr (*p* = .01) were increased significantly (*p* < .05) corresponding to pit ages, and their contents were increased from 1.88 to 2.93 mg/g (Y1–Y50), from 2.50 to 3.75 mg/g (Y1–Y50), from 0.02 to 0.06 mg/g (Y1–Y50), and from 1.09 to 2.12 mg/g (Y1–Y50), respectively. In the lower layers, all the FAAs were increased significantly (*p* < .05) in Y50 compared to that of Y1. Moreover, the lower layer had the highest total FAA content, follow by the middle layer. Some of the microorganisms grown in ZPs have been reported to produce acidic carboxyl proteases. This activity of this enzyme is important in the degradation of proteins and their conversion into amino acids, which in turn enhances microbial growth and the formation of trace levels of aromatic compounds (Shen, 2007). Moreover, the biodiversity of microorganisms in the ZPs of the aged fermentation pit and lower layer is higher than that of the young fermentation pit and the upper layer. Thus, this may lead to increase in the total FAA content in aged fermentation pit of RLs (Y20 and Y50) and lower layer RLs.

Principal component analysis was performed to comparatively analyze the relationships of the FAAs in the RLs of different pit ages. In the PCA score plot, PC1 and PC2 were extracted to be 61.6% and 21.6%, respectively. As shown, the RLs of different fermentation pit ages and different layers were clearly separated and were especially discriminated by PC1 (Figure 4e). The distribution results showed that the FAAs among the different samples had significant differences and could be effectively separated.

### 3.4 Determination of biomarkers in RLs of different fermentation pit ages

**FIGURE 5:**
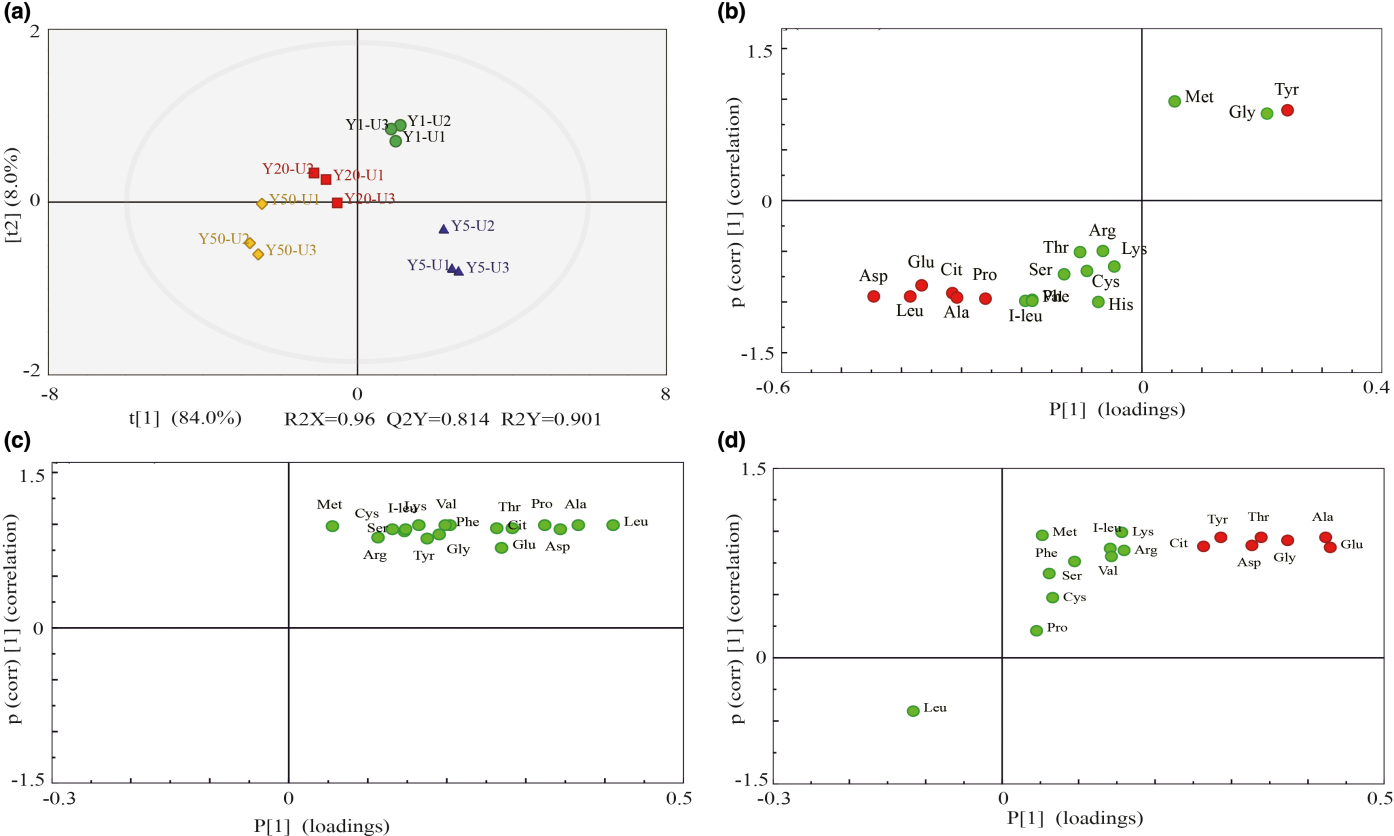
Determination of potential biomarkers about upper‐layer of RLs in different fermentation pit ages. (a) OPLS‐DA score plots of RLs in different fermentation pit ages; (b–d) S‐plot obtained from the OPLS‐DA. Potential biomarkers located in the bottom left and top right of the S‐plot are marked in red

In general, the exact differences between samples cannot be explained through the visual discrimination generated from a PCA algorithm. In the present work, we therefore implemented a supervised classification through an OPLS‐DA model to monitor the degree of transformation of the metabolites in RLs of different fermentation pit ages. The OPLS‐DA can discriminate two or more groups (classes) using multivariate data, and compared to PLS‐DA, OPLS‐DA can filter out noise unrelated to classification information and thereby improve analytical ability, ensuring the validity of the models, and maximizing the differences between the groups within the models (Ai et al., 2021). Besides, the prediction parameters of OPLS‐DA exhibit strong goodness of fit (R2X, R2Y, Q2). Q2 is a very important index for evaluating the models in OPLS‐DA, and the model will be indicated as an excellent one, when it is >0.9 (Wei et al., 2018). In order to further investigate the differences in FAAs and the correlation between samples, twelve RLs were compared in pairs, and the OPLS‐DA model was established to observe the differential FAAs between groups. In the upper layer of RLs, the score‐plot output of the OPLS‐DA multivariate approach is presented in Figure 5a and Figure S1. The parameters for the classifications were observed between Y1 and Y5 (R2X = 0.927, R2Y = 1, Q2 = 0.995), between Y5 and Y20 (R2X = 0.990, R2Y = 1, Q2 = 0.993), and between Y20 and Y50 (R2X = 0.885, R2Y = 0.978, Q2 = 0.924). Besides, the OPLS‐DA score plots showed a high degree of discrimination among the groups of samples, with a clear separation between the RLs samples of different pit ages. Moreover, the S‐plot derived by OPLS‐DA presents “S‐form”, and ions that contributed to the separation were selected from the S‐plot and regarded as potential markers, and they are marked in red (Figure 5b–d); potential markers that decided the differences were also identified by VIP values. Based on VIP >1, there were seven biomarkers between Y1 and Y5 (one upregulated, six downregulated); of them, Tyr was upregulated, Asp, Leu, Glu, Cit, Ala, and Pro were downregulated, compared to Y1. No biomarkers were between Y5 and Y20. Seven biomarkers were upregulated between Y20 and Y50, including Glu, Ala, Gly, Thr, Tyr, and Cit.

**FIGURE 6:**
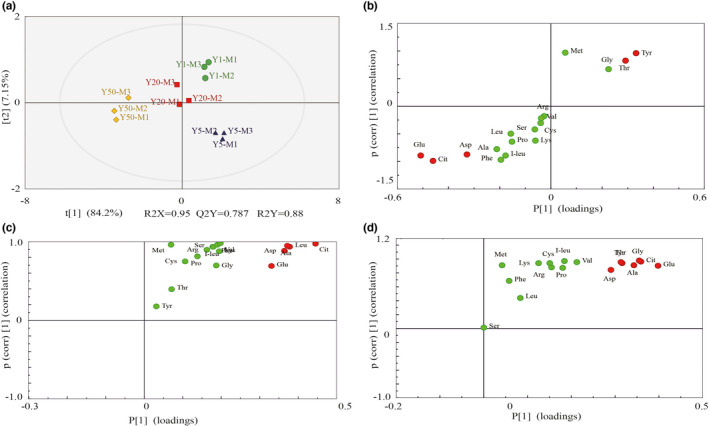
Determination of potential biomarkers about middle‐layer of RLs in different fermentation pit ages. (a) OPLS‐DA score plots of RLs in different fermentation pit ages; (b–d) S‐plot obtained from the OPLS‐DA. Potential biomarkers located in the bottom left and top right of the S‐plot are marked in red

In the middle layer of RLs, excellent model parameters (Y1–Y5: R2X = 0.898, R2Y = 1, Q2 = 0.992; Y5–Y20: R2X = 1, R2Y = 1, Q2 = 1; Y20–Y50: R2X = 0.863, R2Y = 0.999, Q2=0.987) were detected in our experiment (Figure 6a and Figure S2). Besides, cross validation and response permutation testing (RPT) showed that no overfitting occurred in the OPLS‐DA model. Obviously, the OPLS‐DA score plots showed a high degree of discrimination among the groups of samples, with a clear separation between the RLs of different pit ages. Five biomarkers were between Y1 and Y5 (two up‐regulated, three down‐regulated); of them, Tyr and Thr were upregulated; Glu, Cit, and Asp were downregulated comparing with Y1. Five biomarkers were upregulated between Y5 and Y20, including Cit, Leu, Asp, Ala, and Glu. Six biomarkers were upregulated between Y201 and Y50, including Glu, Cit, Gly, Ala, Thr, Tyr, and Asp (Figure 6b,d,f).

**FIGURE 7:**
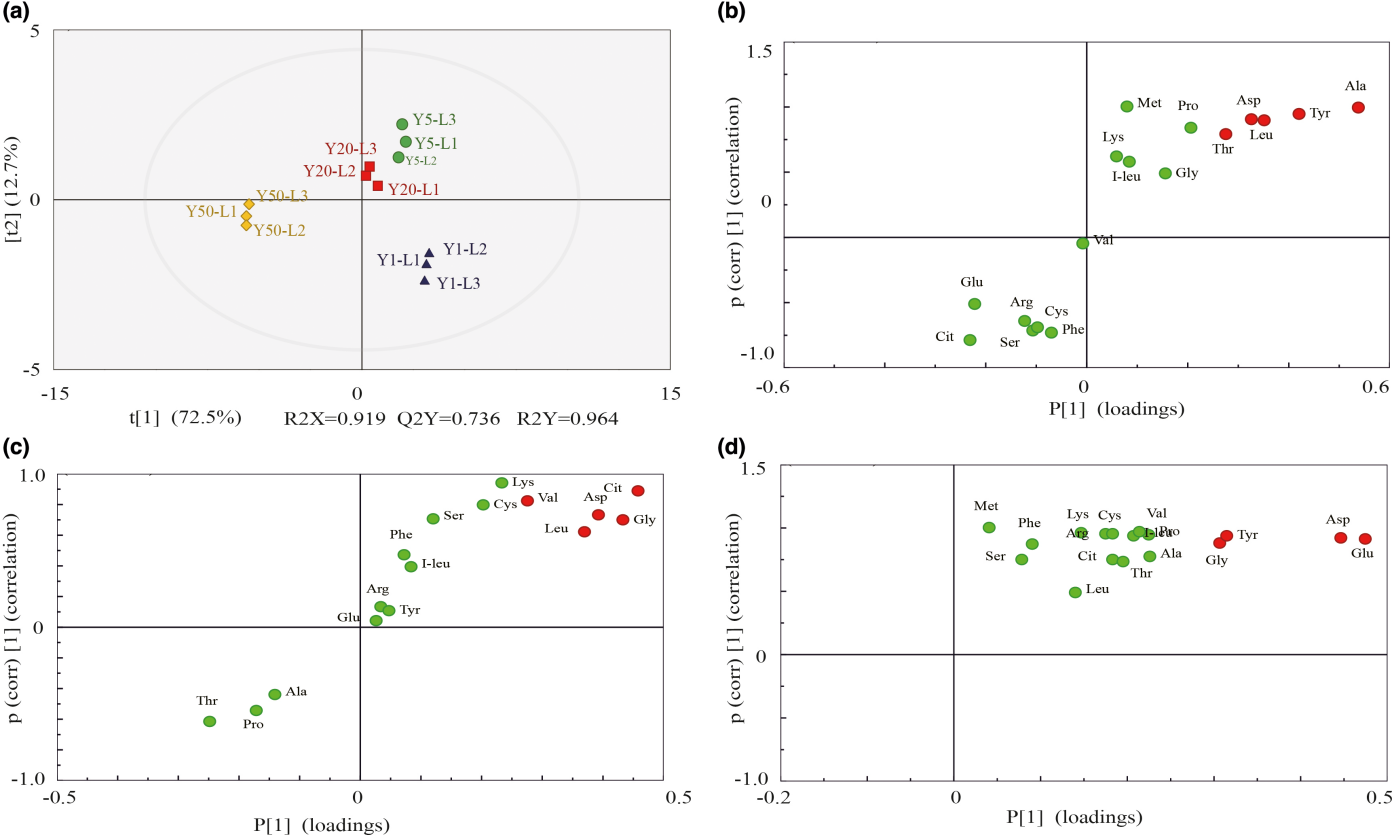
Determination of potential biomarkers about lower‐layer of RLs in different fermentation pit ages. (a) OPLS‐DA score plots of RLs in different fermentation pit ages; (b–d) S‐plot obtained from the OPLS‐DA. Potential biomarkers located in the bottom left and top right of the S‐plot are marked in red

In the lower layer of RLs, the parameters for the classifications were observed between Y1 and Y5 (R2X = 0.898, R2Y = 1, Q2 = 0.992); Y5 and Y20 (R2X = 1, R2Y = 1, Q2 = 1); as well as between Y20 and Y50 (R2X = 0.863, R2Y = 0.999, Q2 = 0.987; Figure 7a and Figure S3). Besides, the OPLS‐DA score plots showed a high degree of discrimination among the groups of samples, with a clear separation between the RLs of different pit ages, indicating major distinctions in the metabolic profiles. Moreover, five biomarkers were upregulated between Y1 and Y5, including Ala, Tyr, Leu, Asp, and Thr. Five biomarkers were upregulated between Y5 and Y20, including Cit, Gly, Asp, Leu, and Val. Four biomarkers were upregulated between Y20 and Y50, including Glu, Asp, Tyr, and Gly (Figure 7b,d,f). The results demonstrated that the OPLS‐DA model had the preferable predication performance and reliability and could be used to explore the metabolic differences in the RLs of different fermentation pit ages. In our study, Glu was the most abundant secondary‐metabolite compound in raw liquor. Glu has been reported to have an influence on the aroma of the Chinese liquor (Wu, 2013), and it may play a major role in the quality of WLFL.

### 3.5 Correlation network analysis between FAAs and microbial communities

**FIGURE 8:**
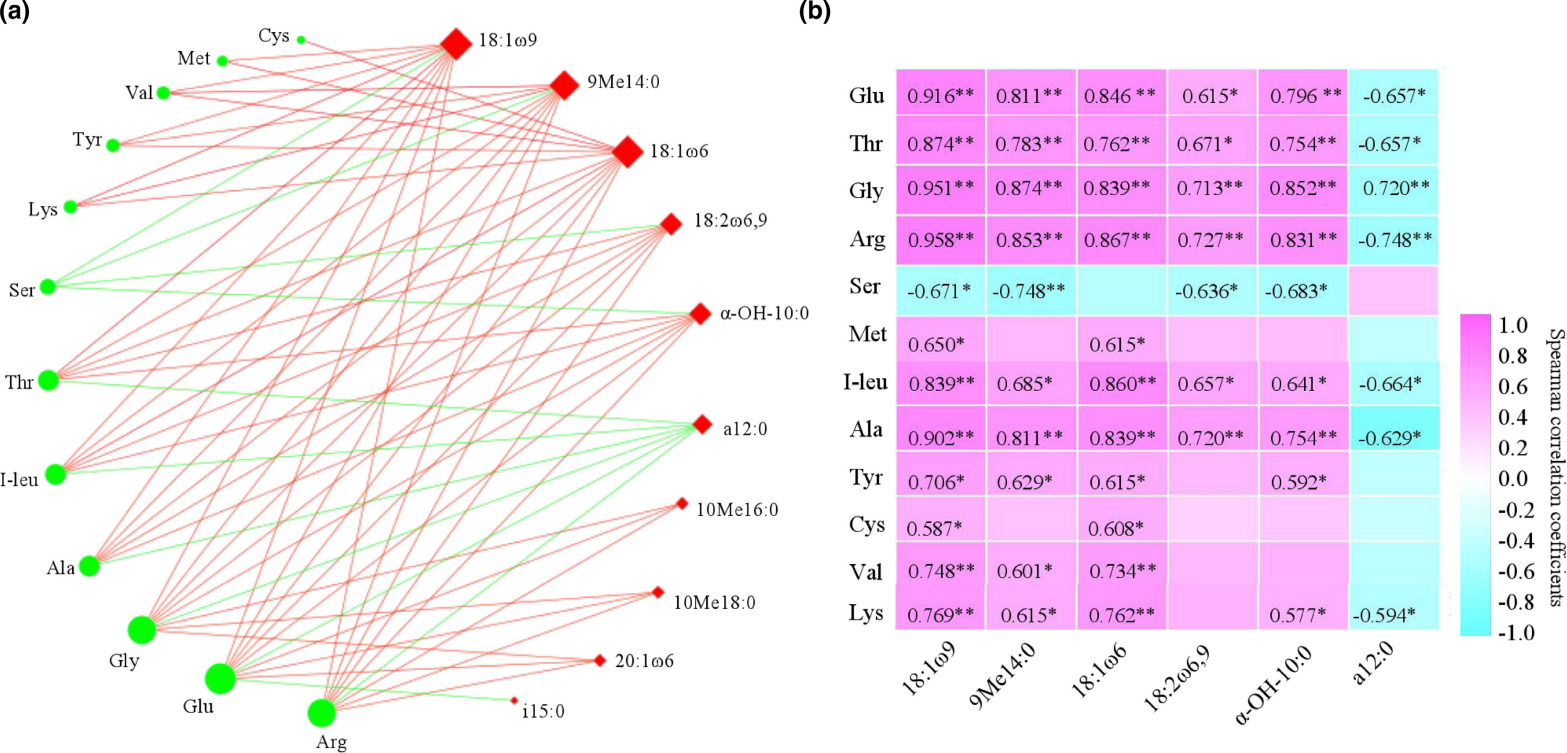
Correlation analysis between microbial communities and FAAs. (a) Correlation network and a connection (i.e., edge) indicates a positive (red line) or negative (green line) correlation with the absolute value of Spearman's correlation coefficient more than 0.6. (b) Spearman correlation coefficients between predominant microorganisms and main FAAs, *p value < 0.05; **p < .01

In order to reveal the relationship between FAAs and microbial communities, we calculated the Spearman correlation coefficients (*ρ*) between the FAAs and microbial communities, and |*ρ*| with >0.6 are shown using a network diagram (Figure 8a and Table S3). As shown in Figure 8, for the co‐occurrence network, 22 effective connection nodes and 62 edges were observed. Among them, the stronger connection nodes (≥6 edges) were mostly distributed in 18:1ω9, 9Me14:0, 18:1ω6, 18:2ω6,9, α‐OH‐10:0, and a12:0, indicating that they may be the dominant microorganisms of ZPs during the fermentation process of WLFL; then, we further calculated the *ρ* between the main FAAs and PLFAs (Figure 8b). Fungal biomarker 18:1ω9 and G^−^'s biomarker 18:1ω6 exhibited significantly positive correlations (red edges) with all FAAs (except of Ser), while G^+^'s biomarker a12:0 was negatively correlated to some FAAs (green edges), including Thr, I‐leu, Ala, Gly, Glu, and Arg. Thus, G^−^, anaerobe, and fungi can be beneficial to form the overall sensorial quality (e.g., flavor and mouthfeel) of RLs in this study, which was consistent with previous reports (Wang et al., 2021; Wu et al., 2017; Zheng et al., 2013). Moreover, actinomycete's biomarkers, 10Mel6:0 and 10Me18:0, and G^−^'s biomarker 20:1ω6 were negatively correlated to Gly, Glu, and Arg (Figure 8). The above results suggested that the ZPs microbial consortia may play more important roles in the FAAs formation of WLFL.

## 4 CONCLUSION

In this study, microbial communities' characteristics and differences of ZPs with different pit ages were elucidated by PLFA. PLFA revealed that bacteria and fungi were included in the ZPs. The total biomass, fungal biomass, and the biomass of G^+^ bacteria increased as the age of the pit increased. The diversity of the microbial community structure and their shift with pit age was explained by PCA and CA analysis. Young ZPs and aged ZPs were well separated from each other, as well as from different layers. Samples from year 1, year 5, year 20, and year 50 of RLs were analyzed using PCA, OPLS‐DA models. Based on OPLS‐DA analysis, twelve RLs can be distinguished according to the contents of the potential biomarkers of FAAs, such as Asp, Leu, Glu, Cit, Ala, Pro, Tyr, Gly, and Thr. More importantly, the differential FAAs were positively correlated with G^−^ and fungi, but negatively correlated with G^+^. Overall, this study uncovered the microbial communities in *zaopeis*, free amino acids in RL, and their correlations for WLFL‐RL production. More research is also needed to determine the main source of FAAs produced in the WLFL brewing process, and traceability research on the microorganisms and enzyme from microorganisms that produce FAAs are of considerable significance for laying a theoretical foundation to stabilize the quality of WLFL and increase WLFL output.

## CONFLICT OF INTEREST

The authors declare that there are no conflicts of interest.

## Supporting information

Fig S1

Fig S2

Fig S3

Table S1

Table S2

Table S3

## Data Availability

The data presented in this study are available on request from the corresponding author.
