# Supplementary figures and images for: The microbial communities in Zaopeis, free amino acids in raw liquor, and their correlations for Wuliangye‐flavor raw liquor production
Source: Food Sci Nutr. 2022 Apr 8;10(8):2681–93. doi: 10.1002/fsn3.2872 (PMC9361440; doi:10.1002/fsn3.2872)

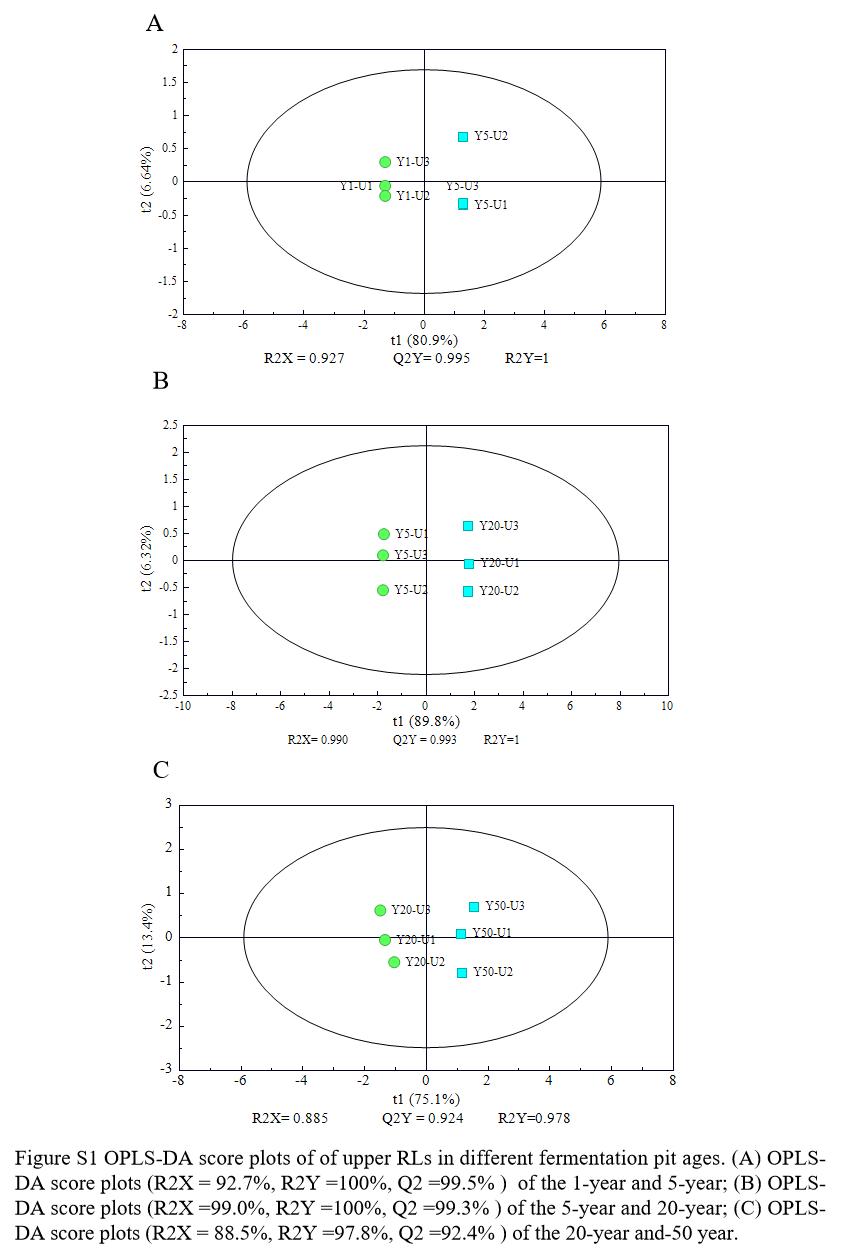

Supplement: Supplementary file 1 — Fig S1 [file FSN3-10-2681-s004.jpg]

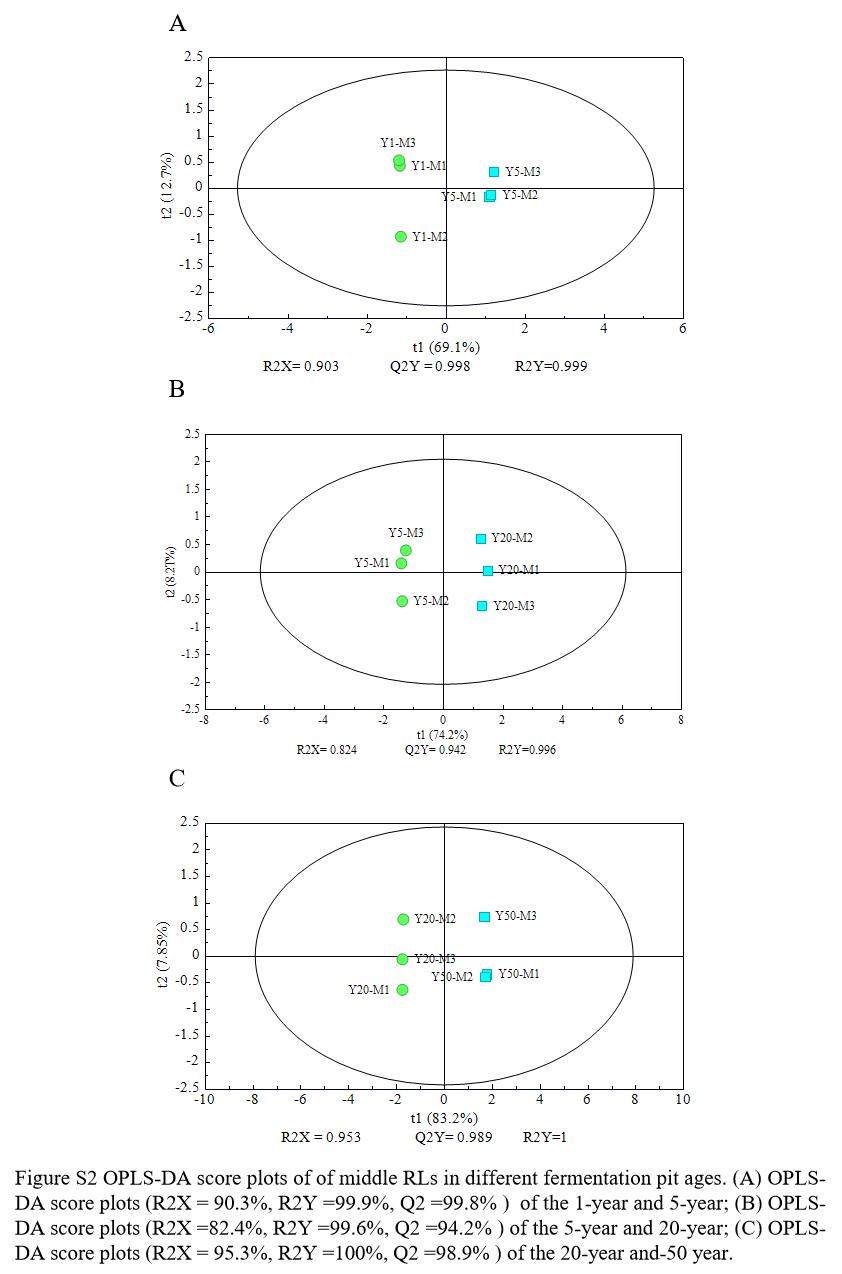

Supplement: Supplementary file 2 — Fig S2 [file FSN3-10-2681-s005.jpg]

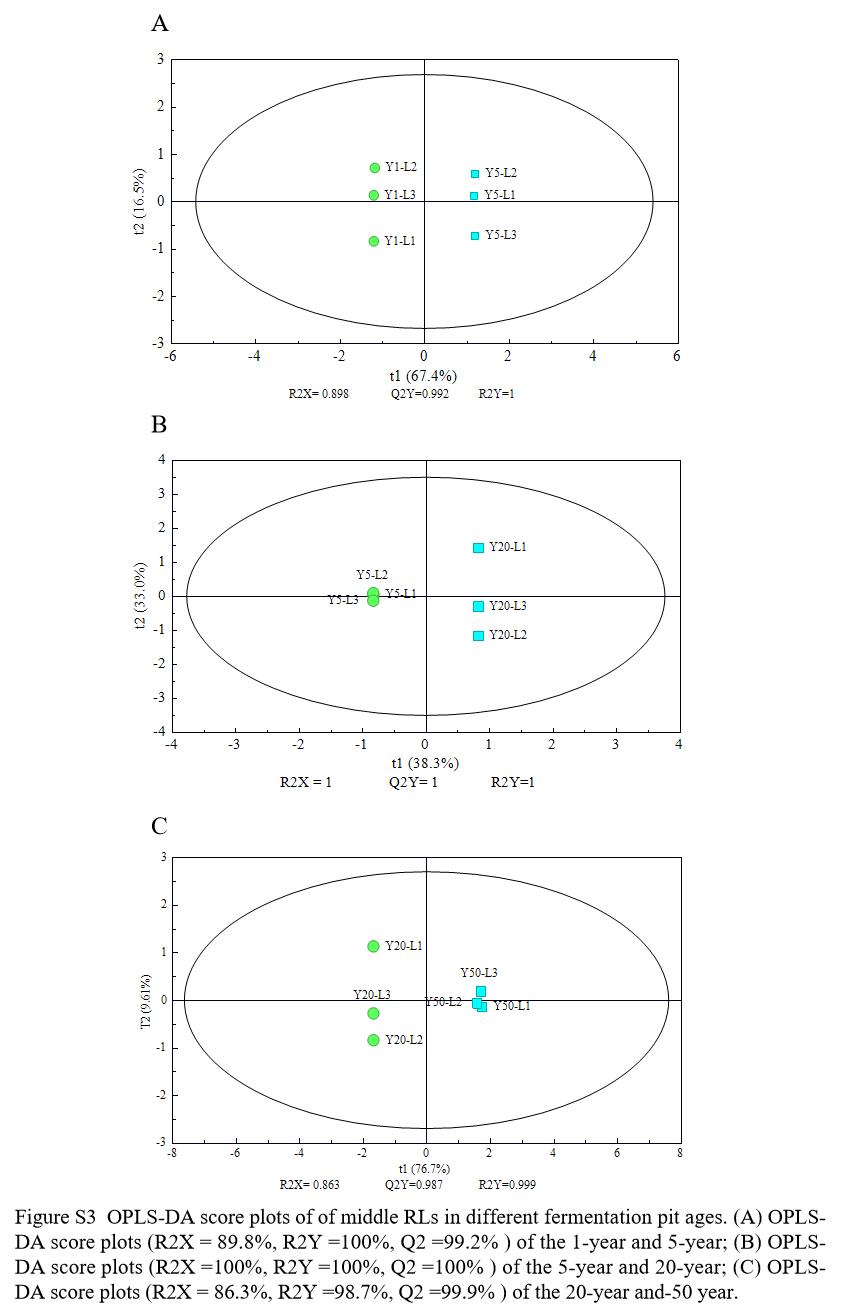

Supplement: Supplementary file 3 — Fig S3 [file FSN3-10-2681-s003.jpg]
