# Supplementary material for: The microbial communities in Zaopeis, free amino acids in raw liquor, and their correlations for Wuliangye‐flavor raw liquor production
Source: Food Sci Nutr. 2022 Apr 8;10(8):2681–93. doi: 10.1002/fsn3.2872 (PMC9361440; doi:10.1002/fsn3.2872)
Supplement: Supplementary file 4 — Table S1 [file FSN3-10-2681-s006.doc]

Table S1-PLFA contents of ZPs at different pit ages, data were means

| **PLFAs** | **Y1-U** | **Y1-M** | **Y1-L** | **Y5-U** | **Y5-M** | **Y5-L** | **Y20-U** | **Y20-M** | **Y20-L** | **Y50-U** | **Y50-M** | **Y50-L** |
| --- | --- | --- | --- | --- | --- | --- | --- | --- | --- | --- | --- | --- |
| a8:0 | 0.00 | 0.12 | 2.79 | 0.00 | 0.14 | 3.39 | 0.00 | 0.16 | 4.02 | 0.00 | 0.19 | 4.12 |
| a9:0 | 0.00 | 0.00 | 0.00 | 0.18 | 0.15 | 0.21 | 0.28 | 0.19 | 0.28 | 0.31 | 0.23 | 0.32 |
| i9:0 | 0.00 | 0.09 | 1.01 | 0.00 | 0.10 | 1.25 | 0.00 | 0.11 | 1.15 | 0.00 | 0.14 | 1.52 |
| α-OH-10:0 | 0.00 | 0.00 | 0.00 | 0.26 | 0.42 | 0.88 | 0.45 | 0.62 | 1.29 | 0.55 | 0.86 | 1.56 |
| a12:0 | 0.23 | 0.20 | 0.17 | 2.42 | 2.16 | 1.80 | 0.12 | 0.10 | 0.08 | 0.16 | 0.10 | 0.10 |
| i13:0 | 0.80 | 0.66 | 0.44 | 0.59 | 0.46 | 0.32 | 1.07 | 0.82 | 0.49 | 1.14 | 0.87 | 0.50 |
| a14:0 | 1.80 | 1.54 | 1.24 | 2.64 | 2.18 | 1.79 | 1.82 | 1.26 | 0.95 | 1.93 | 1.38 | 0.97 |
| a15:0 | 2.44 | 0.86 | 0.11 | 1.57 | 0.41 | 0.00 | 2.52 | 0.82 | 0.15 | 2.74 | 1.05 | 0.15 |
| i15:0 | 0.92 | 0.61 | 0.40 | 0.56 | 0.36 | 0.26 | 0.60 | 0.32 | 0.20 | 0.61 | 0.40 | 0.18 |
| 16:1ω5c | 0.00 | 0.02 | 0.06 | 0.00 | 0.14 | 0.47 | 0.00 | 0.03 | 0.10 | 0.00 | 0.04 | 0.14 |
| i16:0 | 3.40 | 1.71 | 0.42 | 1.30 | 0.65 | 0.16 | 2.66 | 1.08 | 0.25 | 2.66 | 1.12 | 0.23 |
| a16:0 | 2.99 | 1.17 | 0.35 | 1.19 | 0.42 | 0.16 | 2.19 | 0.73 | 0.24 | 2.37 | 0.74 | 0.21 |
| 14:00 | 1.59 | 1.17 | 0.81 | 1.24 | 0.92 | 0.63 | 2.02 | 1.35 | 0.98 | 2.49 | 1.57 | 1.11 |
| 15:00 | 5.63 | 4.37 | 2.22 | 3.13 | 2.62 | 1.23 | 8.46 | 6.19 | 2.84 | 8.81 | 6.56 | 3.56 |
| 10Me16:0 | 0.00 | 0.00 | 0.00 | 0.00 | 0.00 | 0.43 | 0.00 | 0.22 | 0.61 | 0.00 | 0.27 | 0.73 |
| 16:00 | 7.13 | 8.29 | 10.73 | 7.61 | 9.68 | 11.83 | 13.71 | 14.03 | 17.29 | 16.37 | 17.34 | 19.45 |
| 17:00 | 0.11 | 0.25 | 0.31 | 0.00 | 0.00 | 0.00 | 0.59 | 0.87 | 1.00 | 0.67 | 1.11 | 1.38 |
| 18:00 | 1.14 | 1.85 | 2.62 | 0.52 | 0.85 | 1.28 | 2.39 | 3.20 | 4.71 | 2.91 | 4.04 | 5.20 |
| cy17:0 | 0.00 | 0.09 | 0.22 | 0.00 | 0.09 | 0.23 | 0.00 | 0.07 | 0.21 | 0.00 | 0.11 | 0.30 |
| 18:1ω9 | 0.60 | 0.92 | 1.13 | 1.51 | 2.46 | 3.15 | 4.60 | 6.34 | 8.20 | 5.82 | 7.10 | 9.17 |
| 18:1ω6 | 0.18 | 0.32 | 0.39 | 0.26 | 0.41 | 0.14 | 0.65 | 1.05 | 1.22 | 0.81 | 1.26 | 1.66 |
| 10Me18:0 | 0.00 | 0.00 | 0.00 | 0.00 | 0.76 | 1.33 | 0.00 | 1.32 | 2.27 | 0.00 | 1.50 | 2.81 |
| cy19:0 | 4.18 | 9.01 | 12.35 | 4.35 | 9.21 | 13.44 | 5.78 | 10.06 | 14.34 | 6.92 | 12.98 | 16.51 |
| 18:2ω6,9 | 0.22 | 0.24 | 0.35 | 0.07 | 0.08 | 0.12 | 1.20 | 1.14 | 1.77 | 1.54 | 1.41 | 2.21 |
| 20:1ω6 | 0.00 | 0.00 | 0.00 | 0.00 | 0.11 | 0.30 | 0.00 | 0.13 | 0.35 | 0.00 | 0.16 | 0.40 |
| 9Me14:0 | 0.00 | 0.04 | 0.06 | 0.20 | 0.29 | 0.46 | 0.34 | 0.40 | 0.68 | 0.41 | 0.50 | 0.81 |
