# Supplementary material for: The microbial communities in Zaopeis, free amino acids in raw liquor, and their correlations for Wuliangye‐flavor raw liquor production
Source: Food Sci Nutr. 2022 Apr 8;10(8):2681–93. doi: 10.1002/fsn3.2872 (PMC9361440; doi:10.1002/fsn3.2872)
Supplement: Supplementary file 5 — Table S2 [file FSN3-10-2681-s002.doc]

Table S2-The contents of the FAAs of RLs at different fermentation pit ages, data were means

| **FAAs** | **Y1-U** | **Y1-M** | **Y1-L** | **Y5-U** | **Y5-M** | **Y5-L** | **Y20-U** | **Y20-M** | **Y20-L** | **Y50-U** | **Y50-M** | **Y50-L** |
| --- | --- | --- | --- | --- | --- | --- | --- | --- | --- | --- | --- | --- |
| Glu | 4.42 | 5.35 | 5.78 | 3.84 | 4.50 | 5.48 | 4.47 | 5.15 | 5.51 | 5.22 | 6.31 | 7.02 |
| Asp | 2.94 | 3.02 | 2.65 | 2.18 | 2.66 | 3.01 | 3.01 | 3.24 | 3.32 | 3.44 | 3.91 | 4.62 |
| Cit | 2.47 | 2.11 | 1.87 | 2.07 | 1.48 | 1.66 | 2.55 | 2.27 | 2.02 | 2.84 | 3.16 | 2.30 |
| Thr | 1.49 | 1.88 | 2.63 | 1.42 | 2.19 | 2.93 | 1.98 | 2.24 | 2.78 | 2.42 | 2.93 | 3.09 |
| Gly | 1.22 | 2.50 | 3.00 | 1.40 | 2.72 | 3.15 | 1.67 | 2.91 | 3.55 | 2.22 | 3.75 | 4.20 |
| Arg | 0.44 | 0.59 | 0.92 | 0.41 | 0.58 | 0.84 | 0.50 | 0.70 | 0.86 | 0.62 | 0.88 | 1.06 |
| Ser | 0.61 | 0.47 | 0.38 | 0.53 | 0.44 | 0.33 | 0.68 | 0.58 | 0.36 | 0.73 | 0.58 | 0.41 |
| Met | 0.01 | 0.02 | 0.03 | 0.02 | 0.03 | 0.05 | 0.04 | 0.05 | 0.05 | 0.05 | 0.06 | 0.06 |
| Leu | 2.28 | 2.17 | 1.69 | 1.71 | 2.02 | 2.11 | 2.85 | 2.61 | 2.44 | 2.74 | 2.72 | 2.69 |
| Pro | 0.87 | 0.98 | 0.82 | 0.62 | 0.87 | 0.97 | 1.33 | 0.97 | 0.89 | 1.35 | 1.22 | 1.22 |
| I-leu | 0.44 | 0.48 | 0.54 | 0.30 | 0.38 | 0.58 | 0.45 | 0.52 | 0.61 | 0.53 | 0.75 | 0.88 |
| Ala | 1.42 | 2.43 | 3.13 | 1.06 | 2.27 | 4.03 | 1.97 | 2.86 | 3.96 | 2.66 | 3.69 | 4.36 |
| Tyr | 0.92 | 1.09 | 1.17 | 1.15 | 1.43 | 1.74 | 1.39 | 1.44 | 1.77 | 1.70 | 2.12 | 2.42 |
| Cys | 0.39 | 0.41 | 0.45 | 0.35 | 0.40 | 0.41 | 0.47 | 0.46 | 0.48 | 0.50 | 0.62 | 0.70 |
| Val | 0.61 | 0.70 | 0.83 | 0.49 | 0.68 | 0.83 | 0.77 | 0.85 | 0.96 | 0.85 | 1.16 | 1.26 |
| His | 0.02 | 0.00 | 0.00 | 0.00 | 0.00 | 0.00 | 0.00 | 0.00 | 0.00 | 0.00 | 0.00 | 0.00 |
| Phe | 0.30 | 0.32 | 0.28 | 0.18 | 0.20 | 0.26 | 0.44 | 0.36 | 0.28 | 0.47 | 0.39 | 0.34 |
| Lys | 0.11 | 0.24 | 0.29 | 0.10 | 0.22 | 0.31 | 0.28 | 0.38 | 0.39 | 0.37 | 0.49 | 0.53 |
