# Supplementary material for: The microbial communities in Zaopeis, free amino acids in raw liquor, and their correlations for Wuliangye‐flavor raw liquor production
Source: Food Sci Nutr. 2022 Apr 8;10(8):2681–93. doi: 10.1002/fsn3.2872 (PMC9361440; doi:10.1002/fsn3.2872)
Supplement: Supplementary file 6 — Table S3 [file FSN3-10-2681-s001.doc]

**Table S3-**Spearman correlation coefficients between predominant microbes and main FAAs

| **FAAs** | **18:1ω9** | **9Me14:0** | **18:1ω6** | **18:2ω6,9** | **α-OH-10:0** | **a12:0** |
| --- | --- | --- | --- | --- | --- | --- |
| Glu | 0.916** | 0.811** | 0.846** | 0.615* | 0.796** | -0.657* |
| Asp | 0.462 | 0.315 | 0.497 | 0.175 | 0.303 | -0.203 |
| Cit | -0.252 | -0.448 | -0.042 | -0.245 | -0.408 | 0.042 |
| Thr | 0.874** | 0.783** | 0.762** | 0.671* | .754** | -0.657* |
| Gly | 0.951** | 0.874** | 0.839** | 0.713** | 0.852** | -0.720** |
| Arg | 0.958** | 0.853** | 0.867** | 0.727** | 0.831** | -0.748** |
| Ser | -.0671* | -0.748** | -0.573 | -0.636* | -0.683* | 0.441 |
| Met | 0.650* | 0.531 | 0.615* | 0.469 | 0.486 | -0.476 |
| Leu | -0.049 | -0.182 | 0.084 | -0.105 | -0.211 | 0.028 |
| Pro | 0.098 | 0.014 | 0.140 | -0.112 | -0.035 | 0.133 |
| I-leu | 0.839** | 0.685* | 0.860** | 0.657* | 0.641* | -0.664* |
| Ala | 0.902** | 0.811** | 0.839** | 0.720** | 0.754** | -0.629* |
| Tyr | 0.706* | 0.629* | 0.615* | 0.510 | 0.592* | -0.490 |
| Cys | 0.587* | 0.448 | 0.608* | 0.329 | 0.416 | -0.427 |
| Val | 0.748** | 0.601* | 0.734** | 0.510 | 0.563 | -0.545 |
| His | -0.480 | -0.480 | -0.393 | -0.218 | -0.396 | 0.218 |
| Phe | -0.021 | -0.182 | 0.077 | -0.147 | -0.183 | 0.007 |
| Lys | 0.769** | 0.615* | 0.762** | 0.559 | 0.577* | -0.594* |
